# The Change From Passive to Active Parental Consent on Sample Composition; Do We Exclude At-Risk Youth?

**DOI:** 10.1177/0193841X261446673

**Published:** 2026-04-21

**Authors:** Ina M. Koning, Vincent G. van der Rijst

**Affiliations:** 1 Clinical Child and Family studies, 1190 Vrije Universiteit Amsterdam, Amsterdam, The Netherlands; 2 26063 Alcohol- and drugprevention, Trimbos Insititute, Utrecht, The Netherlands

**Keywords:** passive-active parental consent, adolescents, substance use, sample composition

## Abstract

The aim of this study was to investigate the impact of the shift in recent years from passive to active parental consent for youth’ participation in research. A longitudinal study that took place during this change provided the unique opportunity to analyze which adolescent (demographic, alcohol use, norms) and parental factors predict the recipient of consent. The sample consisted of 691 adolescents between 12 and 17 years old (*M*_*age*_ = 14.22; *SD* = 1.03; 44.9% boys). Factors for each and across five domains (socio-demographic, alcohol use, individual, group/peers, and parental factors) were included in a multiple logistic regression analysis to predict non-consent at follow-up. Across domains, results showed that adolescents who are older (*OR* = 0.60, *p* < .001), female (*OR* = 0.60, *p* < .001) and those perceived less strict rules about alcohol (*OR* = 1.26, *p* = .05), have a higher odds of not having consent for participation. These findings indicate that a specific selection of adolescents were given permission to participate in research, yet this was not particularly an at-risk group. Implications of these findings, such as the balance between autonomy and protection, are discussed.

Underage youth are often a subject of research in the field of wellbeing and risk behaviors. Parental consent is an important ethical and legal consideration in research involving underage population. There are two main procedures commonly used to obtain informed authorization from the guardians: active and passive consent. In both cases, researchers inform parents about the study and ask their permission for their child’s participation. Active consent requires parents to reply, usually through a signed letter, giving their active permission for their child to participate. In contrast, in passive consent procedures, permission is assumed, unless parents reply informing that they do not want their child to participate. Recently, due to changes in privacy regulations, a shift has taken place from obtaining passive consent to active consent, which may have implications for the sample included. Hence, it is important to analyze what impact this change has on the included sample under investigation and its subsequent representativeness.

Previously, depending on the type of research, active (e.g., medical research) as well as passive (e.g., research on non-invasive topics) parental consent methods could be authorized by ethics review boards. Nevertheless, in May 2018, the General Data Protection Regulation (GDPR) from the European Commission entered into force, determining that “children merit specific protection with regard to their personal data, as they may be less aware of the risks, consequences and safeguards concerned and their rights in relation to the processing of personal data” (EU, 2018, recital 18). Moreover, the GDPR establishes in article 8 “Where the child is below the age of 16 years, such processing shall be lawful only if and to the extent that consent is given or authorized by the holder of parental responsibility over the child.” Although the type of consent is not mentioned in the regulation, it is established that it should be given through a clear affirmative act, leading to ethical review boards to urge researchers to use active consent procedures over passive consent nowadays. This shift from passive to active parental consent may have implications for the characteristics of youth and parents that are allowed to participate in research.

One of the problems with the drift from passive to active consent is that when requirements for consent become stricter, participation rates become lower (Anderman et al., 1995; Dent et al., 1993; Pokorny et al., 2001; Tigges, 2003; Villarreal et al., 2023), with a risk of bias in terms of the sample characteristics as well as on important dependent variables (Pokorny et al., 2001). Particularly for research on risk behavior in adolescence -such as drinking, smoking or sexual activity—active parental consent might jeopardize the correct assessment of the needs of the populations or silence the voices of those who are most in need of an intervention (Baker et al., 2001; Rojas et al., 2008; Unger et al., 2004). For example, Pokorny et al. (2001) demonstrated that requiring active parental consent resulted in lower rates of participation, with those adolescents participating being more likely to be younger and female and reported lower rates of lifetime tobacco use (not alcohol use). Consequently, there is a need to analyze the changes in the composition of the sample while shifting from passive to active consent procedures.

Previous research has shown that there is an association between getting active parental consent and perceiving higher parental interest by the participants (Jelsma et al., 2012). Therefore, it can be argued that the parent–child relationship, as well as the level of parental involvement in their child’s life, can predict if the child will get active parental consent or not. Coleman (1988) defines the *familial social capital* primarily as the relationship between children and parents, this intergenerational closeness can define the resources the child can access and the benefits they can obtain. A broader definition of social capital also considered the resources a group or family can obtain through their social network (Moore & Kawachi, 2017). For example, access to the information necessary to give active parental consent. It has been established that the deprivation of social capital leads to health inequalities (Moore et al., 2013) and can act as a catalyzer of youth risk behavior, like alcohol consumption (McPherson et al., 2014). For example, in their study on parents’ willingness to participate in psychological studies and to support their children’s participation, Jungmann et al. (2023) identified parental uncertainty and lack of interest as key barriers specifically to supporting their children’s involvement. However, the association with different types of consent remains unstudied.

Few studies have empirically investigated the sample implications of changing from passive to active consent. A meta-analysis conducted by Liu and colleagues (2017), including 11 studies, demonstrated that response rates for active consent groups were significantly lower compared to passive consent groups: 30–60% and 90–100%, respectively. A recent meta-analysis among 38 studies on school-based mental health screening also demonstrated similar participation rates; 55–58% for active consent and 90-96% for passive consent (Villarreal et al., 2023). This is supported by the study of Tigges (2003) who demonstrated similar response rates for studies on parental consent and adolescent risk behavior research. Also, using active parental consent can introduce sampling bias (White et al., 2004). For example, men and older adolescents, ethnic minority groups and adolescents with lower grades were underrepresented in studies with active parental consent (Anderman et al., 1995; Esbensen et al., 1999; Kearney et al., 1983; Tigges, 2003), as well as less-educated parents or families with one-household parent in some (Severson & Ary, 1983) yet not all studies (Pokorny et al., 2001). Several authors have argued that the difference in family social capital is influenced by sociodemographic characteristics like gender, ethnicity, or level of education (Morgan, 2011; White, 2008). Aside from the possible difference in social capital, the previously mentioned demographic characteristics also place adolescents in the higher-risk group of substance use (Evans-Polce et al., 2016), so their exclusion from an active consent sample might lead to misrepresentation bias.

One of the crucial points that have been documented regarding differences in the active and passive consent samples is the prevalence of risk factors associated with risk behavior. Adolescents who did not obtain active parental consent were less likely to be involved in extracurricular activities (Anderman et al., 1995), and they were more impulsive and tended to report less prosocial attitudes and positive peer interactions (Dent et al., 1993; Esbensen et al., 1999). These findings are consistent with the ones suggesting that the same group reported significantly higher numbers of substance use, including alcohol, tobacco, marijuana, and other illegal drugs (Anderman et al., 1995; Dent et al., 1993; Esbensen et al., 1999; Pokorny et al., 2001; Severson & Ary, 1983). In relationship with drinking practices, students in the passive consent group were significantly more likely to report drinking in the previous week (White et al., 2004) and being in the high-risk drinking group, that measured the amount of drinking and negative consequences of excessive drinking (Frissell et al., 2004).

One possible explanation of adolescents who did not receive active parental consent reporting more risk factors, is precisely the lack of family social capital. Having a closer family relationship can act as a protective factor of high-risk activities like substance abuse (Pettit et al., 2011). Richardson and colleagues (1989) argued that the child’s perception of a lack of parental involvement might lead to an expression of this autonomy through high risks behaviors as an effort to achieve more attention. Furthermore, the presence of social capital deprivation understood as negative social relationships that act as risk rather than protective factors, including parent-child conflict, peer pressure, or the absence of prosocial relationships (Evans-Polce et al., 2016), can also explain the association between not receiving active parental consent and being from a higher at-risk group of adolescents.

The current research is part of a larger longitudinal school-based project that examines alcohol related risk factors and youth behaviors, as an effort to build evidence-based interventions and prevention strategies. This study examined the implications of changing from passive to active parental consent procedures, and the repercussions that might have on the accurate assessment of the sample characteristics and alcohol related behaviors. The purpose of this study is to enlarge the existing literature, by analyzing not only the differences in sample characteristics, but also the different mechanisms the adolescents and their families used to cope with alcohol use, based on their family social capital. The exclusion of students that did not present active parental consent might lead to an erroneous assessment of the needs of the population, misleading the course and evaluation of existing and future interventions.

## Methods

### Procedure

Program LEF is a quasi-experimental study in the Netherlands including two high schools (experimental and control condition) that aims to delay the onset of alcohol use among underaged youth (Koning et al., 2021). Data were collected by trained research assistants in classrooms using online questionnaires, available on a secured website. Four waves of data collection has taken place, where in the first three waves passive parental consent was obtained and in the fourth wave this was changed into active parental consent in the experimental school only. At the start of the program (2018), ethical consent was provided to achieve passive parental consent. However, due to stricter regulations in the privacy of participants in research, after the start of the study, active parental consent was encouraged. To investigate the change of type of parental consent between wave 3 and 4, the original wave 3 (now referred to as T1) and wave 4 (now referred to as T2) were included in this study.

At T1, parents received a letter of consent, which informed them about the participation of the school in the program and they were given the opportunity to refuse participation of their child (passive consent). However, in between T1 (February 2019) and T2 (February 2020), the law on stricter privacy procedures (literally translated as “General Data Protection Regulation”) was encouraged to apply in research in the Netherlands. Because of this, the experimental school (and not the control school) preferred to change from passive to active parental consent. Active consent was obtained by informing parents about the study and asking them to indicate in their online student monitoring system to agree or not agree to participation of their child in the study (filling out a self-report survey). In addition, adolescents aged 16 years and older were verbally informed about the study and the voluntary basis of participation.

The study was approved by the Ethics Review Board of the Faculty of Behavioral & Social Sciences at (FETC18-060).

### Sample

A total of 987 adolescents participated in the study at T1 while still using passive consent. Students who would not be able to participate in the next wave anyway (i.e., because they would leave school after graduation) were removed from this sample (*N* = 296). The remaining sample consisted of 691 adolescents between 12 and 17 years old (*M*_*age*_ = 14.22; *SD* = 1.03) and consisted of 44.9% boys. Because the socio-demographic characteristics also are outcome variables, the distribution of the characteristics at individual level for both the non-consent and consent group will be presented later (see also Table 1).

**Table 1.** Percentages, Means and Standard Deviations of Model Variables For The Total Sample, and For The Non-Consent And Consent Group

	**Total(*N* = 691)**	**Non-consent(*N* = 339)**	**Consent(*N* = 352)**
Demographics			
Age (*M*)	14.22 (1.03)	14.54 (1.11)	13.91 (0.85)^***^
Gender: male (%)	44.9	38.8	51.1^**^
Educational level: low (%)	36.8	31.3	42.0^**^
Alcohol use			
Frequency of monthly alcohol use	0.77 (2.12)	1.11 (2.58)	0.44 (1.49)^***^
Weekly drinking	0.86 (2.65)	1.29 (3.23)	0.45 (1.85)^***^
Individual factors			
Self-control	3.58 (0.60)	3.60 (0.64)	3.57 (0.55)
Empowerment	3.80 (0.73)	3.80 (0.79)	3.81 (0.68)
Norms and group factors			
Attitudes about youth drinking	1.88 (1.07)	2.07 (1.17)	1.69 (0.92)^***^
Descriptive norms	2.29 (1.28)	2.56 (1.37)	2.03 (1.12)^***^
Injunctive norms	2.43 (1.97)	2.82 (2.17)	2.06 (1.68)^***^
Group pressure	1.64 (0.66)	1.64 (0.66)	1.64 (0.65)
Parental factors			
Rules about alcohol	4.47 (0.92)	4.27 (1.07)	4.67 (0.69)^***^
Family support	6.10 (1.21)	6.05 (1.30)	6.16 (1.13)
Relatedness	5.17 (1.08)	5.20 (1.07)	5.14 (1.09)
Autonomy	4.96 (1.08)	5.03 (1.10)	4.89 (1.06)
Psychological control	1.95 (0.79)	1.95 (0.80)	1.96 (0.78)

^*^Significantly different from non-consent group at *p* < .05.

^**^Significantly different from non-consent group at *p* < .01.

^***^Significantly different from non-consent group at *p* < .001.

### Measures

The main outcome is whether or not an adolescent has consent to participate in the study. Adolescents at T1 are divided into 0 = no consent and 1 = consent based on their participation in T2. Those adolescents who did not participate at T2 did not receive consent from their parents to participate and/or decided themselves to not participate (adolescents aged 16+).

Multiple factors can describe these groups, divided into five domains: socio-demographic, alcohol use, individual, group/peers, and parental factors.

#### Socio-Demographic

The socio-demographic characteristics used are *age* (continuous), *gender* (0 = boy, 1 = girl), and educational level. *Educational level* was measured on a 6-point scale (ranging from 1 = lower secondary vocational education to 6-pre-university education) and was dichotomized into low (0 = all types of lower secondary vocational education) and high (1 = higher general secondary and pre-university education) education.

#### Alcohol Use

*Frequency of monthly alcohol use* was measured by asking adolescents how often they had drunk alcohol (minimum of one glass) in the last month, indicated from zero to 40 or more on a 14-point scale (O’Malley et al., 1983).

*Weekly drinking* was measured by using the quantity-frequency measure (Engels et al., 1999; Engels & Knibbe, 2000). Frequency was measured by asking the number of days the adolescents usually drink alcohol on weekly basis, while quantity was measured by asking how many glasses of alcohol adolescents usually drinks on a typical day they drink alcohol (9-point scale; 0 = “I don’t drink alcohol” to 8 = “11 glasses or more”). The quantity-frequency was computed by calculating the product of the number of days and the number of glasses, where higher scores indicated more weekly drinking.

#### Individual Factors

*Self-control* reflects the ability to control responses, interrupt undesired behavior, and refrain from acting on them. The measure was the shorter version of the original measure (Tangney et al., 2004), and consisted of 13 items, which were scored on a 5-point scale (1 = “not at all like me” to 5 = “very much like me”). Sample items were “I have trouble saying no” and “I do certain things that are bad for me if they are fun.” Some items were recoded and a mean score was calculated, where a higher score indicated more self-control. Cronbach’s alpha was .79.

*Empowerment* reflects a process of reinforcement in which individuals get a grip on their own situation and environment. It was measured by using the EMPO 2.0 (Damen & Veerman, 2011), consisting of 16 items, which were scored on a 5-point scale (1 = “totally disagree” to 5 = “totally agree”). Sample items are “I am very pleased about how things are going” and “I am confident about the future”. A mean score was calculated, where a higher score indicated more empowerment. Cronbach’s alpha was .95.

#### Norms and Group Pressure

*Attitudes about youth drinking* reflect the acceptability of adolescents consuming alcohol in various situations (e.g., at home, and a party with friends). Participants were asked to what degree they thought it is acceptable for a person of the same age to drink alcohol in various situations (1 = “not acceptable at all” to 5 = “very acceptable”) (Van der Vorst et al., 2006). Originally it contained seven items, in this study we used five items. A mean score was calculated, where a higher score indicated more positive attitudes about youth drinking. Cronbach’s alpha was .93.

*Descriptive norms* reflect the individual’s perceptions of their peers’ behaviors, and this measurement originally consisted of various items measuring several peer substance use behaviors (Elek et al., 2006). In this study, we only used the item regarding alcohol use, by asking participants to estimate how many peers of their age drink alcohol at least once a week (1 = “none of them” to 5 = “all of them”).

*Injunctive norms* reflect the individual’s perceptions of how acceptable their peers find a behavior (Baer, 1994). Participants were asked to what degree they thought that their friends would accept that they would drink alcohol every weekend (1 = “strongly disapprove” to 7 = “strongly approve”).

*Group pressure* reflects the degree to which adolescents experience pressure from their peers to participate in certain behaviors. This was measured by six statements, all starting with “Some adolescents do certain things that they otherwise would not do, because they….” Sample items were “… otherwise would not be a part of the group anymore” and “… are being challenged by their friends.” Participants then were asked to indicate for all six statements to what extent it applied to them on a 5-point scale (1 = ‘does not apply to me at all’ to 5 = “does often apply to me”). A mean score was calculated, where a higher score indicated higher (sensitivity to) peer pressure. Cronbach’s alpha was .87.

#### Parental Factors

*Rules about alcohol* reflect the degree of parental rule-setting regarding alcohol use as perceived by the adolescent (Van der Vorst et al., 2005). Adolescents were asked to what extent their parents approve of them drinking alcohol in various situations. Originally it contained ten items; in this study, we used three items reflecting normative drinking situations (i.e., one glass at home with parents present; at a party with friends; and during the weekend), as suggested by Trager et al. (2021). The items were scored on a 5-point scale (1 = ‘never’ to 5 = ‘always’), and all items were recoded before a mean score was calculated. Higher scores indicated stricter parental rules about alcohol use. Cronbach’s alpha was .93.

*Family support* reflects the degree of social support adolescents experience within their family. In this study, we used the family subscale of the “Multidimensional Scale of Perceived Social Support” (Zimet et al., 1988), with sample items like “I can talk about my problems with my family.” This measure consists of four items, which were scored on a 7-point scale (1 = “very strongly disagree” to 7 = “very strongly agree”). A mean score was calculated, where a higher score indicated more family support. Cronbach’s alpha was .92.

*Relatedness* reflects to what extent adolescents have a meaningful relationship and interaction with their parents, while *autonomy* reflects the need for adolescents to feel like they have control over their own decisions and behavior, independent of their parents. Both concepts were measured based on work of Coskan (2016). Sample items are “My relationship with my parents is an important part of who I am” (relatedness) and “I usually find it comforting if my parents choose in my place what is good for me” (autonomy). Both relatedness and autonomy consisted of six items, which were scored on a 7-point scale (1 = “totally disagree” to 7 = “totally agree”). Some items were recoded and mean scores were calculated for relatedness and autonomy separately. Higher scores indicated more relatedness with parents and a stronger autonomy from their parents, respectively. Cronbach’s alphas were .74 for relatedness, and .68 for autonomy.

*Parental psychological control* reflects the control attempts of parents to constraint, invalidate and manipulate the psychological and emotional experience and expression of their child. Participants were asked to indicate to what extent their parents use psychological control onto them, that is, “My parents always try to change how I feel or think about things.” The instrument is based on work of Barber (1996), consisting of eight items, which were scored on a 5-point scale (1 = “does not apply at all” to 5 = “does totally apply”). A mean score was calculated, where higher scores indicated a stronger perceived psychological control. Cronbach’s alpha was .86.

### Strategy of Analysis

Descriptive statistics were retrieved for all factors for the total group, and for the non-consent and consent group separately, including significance test of group differences. We used multiple logistic regression analysis to test which factors at T1 contribute to a higher chance of participation at T2; the group of adolescents that received parental consent (1 = consent). First, we tested the effects of the factors at T1 on the dummy coded consent variable at T2 separately per domain. Then, all significant factors of each domain were added in one final model, including significant factors across domains. SPSS 26 (IBM, 2019) was used in all analyses.

### Transparency and Openness

We report how we determined our sample size, all data exclusions (if any), all manipulations, and all measures in the study, and we follow Journal Article Reporting Standards (Kazak, 2018). All data, analysis code, and research materials are available by emailing the corresponding author. Data were analyzed using SPSS 26 (IBM, 2019). This study’s design and its analysis were not pre-registered.

## Results

### Consent for Participation

Descriptive analyses showed that 50.9% (*N* = 352) of adolescents who participated at T1 had received active consent to participate in the study at T2. Participants who did not provide active consent (49.1%) were older (*t* = 8.36, *p* < .001), more often female (χ^2^ = 11.42, *p* = .001), and in higher education (χ^2^ = 8.63, *p* = .003). Furthermore, adolescents without active consent drunk more (often) alcohol, reported more positive norms about alcohol, and perceived less strict rules about alcohol compared to adolescents who did have consent for participation (see Table 1). For the other factors, no significant differences between the consent and non-consent group were found.

### Logistic Regression Analysis per Domain

Table 2 presents the estimated odds ratios for the separate analysis per domain.

**Table 2.** Multiple Logistic Regression Analysis Per Domain on Adolescents’ Consent For Participation (Reference Group is Non-Consent)

	**OR**	**99% CI**
Demographics		
Age	0.54***	0.46–0.64
Gender [1 = female]	0.55***	0.40–0.76
Educational level [1 = higher education]	0.82	0.58–1.15
Alcohol use		
Frequency of monthly alcohol use	0.92	0.80–1.05
Weekly drinking	0.91	0.81–1.03
Individual factors		
Self-control	0.90	0.67–1.21
Empowerment	1.05	0.83–1.34
Norms and group factors		
Attitudes about youth drinking	0.86	0.72–1.04
Descriptive norms	0.82*	0.70–0.95
Injunctive norms	0.93	0.83–1.04
pressure	1.07	0.85–1.36
Parental factors		
Rules about alcohol	1.73***	1.43–2.09
Family support	1.17	0.99–1.37
Relatedness	0.81*	0.67–0.98
Autonomy	0.94	0.81–1.09
Psychological control	1.01	0.80–1.28

*Note.* Factors per domain are tested in separate models.

^*^p < .05,

^***^p < .001.

In the *socio-demographic domain*, we found age (OR = 0.54, *p* < .001) and gender (OR = 0.55, *p* < .001) significantly increase the odds to have consent: adolescents who did not have consent were older and more often female compared to adolescents who did have consent. In contrast, educational level did not significantly predict the type of consent group.

For *alcohol use*, and in the *individual domain*, none of the factors contributed significantly in predicting the type of consent group.

One factor in the *norms and group domain* significantly predicted the type of consent; descriptive norms (OR = 0.82, *p* = .01). That is, adolescents reporting to have more peers who drink alcohol on a weekly basis have a higher odds of having no consent. All other factors within this domain were not of significant importance.

Two factors in the *parental domain* were significantly associated with type of consent; rules about alcohol (OR = 1.57, *p* < .001), and relatedness with parents (OR = 0.81, *p* = .03). That is, adolescents who perceived less strict rules about alcohol, and reported more relatedness with their parents were more likely to have no consent to participate. All other factors within this domain were not of significant importance.

### Logistic Analysis Across Domains

In the final model (model fit: *Chi*^*2*^ = 86.9(5), *p* < .001) all significant factors per domain were added in one model. This demonstrated that both demographic characteristics (age and gender, both with an odds ratio of 0.60 (*p* < .001)) remained to be significant predictors of type of consent. That is, adolescents who are older and female have a higher odds of not having consent for participation. Furthermore, although borderline, only rules about alcohol (OR = 1.26, *p* = .05) significantly predicted type of consent; that is, adolescents who perceived less strict rules about alcohol have a higher odds of not having consent for participation. In this final model, descriptive norms and relatedness were no longer significant predictors of type of consent (see also Table 3).

**Table 3.** Multiple Logistic Regression Analysis Including Significant Variables Across Domains On Adolescents’ Consent for Participation (Reference Group is NonConsent)

	**OR**	**99% CI**
Age	0.60***	0.49–0.72
Gender [1 = female]	0.60**	0.43–0.83
Descriptive norms	0.92	0.79–1.06
Rules about alcohol	1.26*	1.01–1.57
Relatedness	0.94	0.80–1.09

^*^p < .05,

^**^p < .01,

^***^p < .001.

## Discussion

The current study investigated the impact of the shift in recent years from passive to active parental consent for youth’ participation in research. A longitudinal study that took place during this change provided the unique opportunity to analyze which adolescent (demographic, alcohol use, norms) and parental factors predict the recipient of consent. The results confirmed that a specific selection of adolescents were given permission to participate in research, yet this was not particularly an at-risk group. Implications of these findings are discussed.

Obtaining active consent for youth’s participation in research, that is, requiring parents to actively provide permission to allow their child to participate in research, resulted in only half of the parents providing this permission. This is a large reduction in the response rate compared to the passive informed consent method, where parents only undertake action if their child is NOT allowed to participate, which often has a response rate of >95% (Koning et al., 2010, 2021; Liu et al., 2017; Villarreal et al., 2023). This demonstrates that the shift from passive to active parental consent has a major impact on the response rate in research involving (underage) youth and subsequent sample composition. Balancing adolescent and family rights with the need for representative data is essential. Passive or self-consent models may be appropriate when information is age-appropriate, the process is low-stress, and the study poses minimal risk (APA, 2018).

Despite the decrease in consent rate, a response rate of 50.9% that was obtained in the current study is actually on the higher end of the range based on the meta-analysis of Liu et al. (2017), who demonstrated response rates between 30 and 60% for the active consent method. The way active parental consent has been obtained in this study may have contributed to this relatively high response rate. That is, parents were asked to provide consent for participation of their child in research activities in general, including the current study as an example, along with other permission topics (e.g., publication of pictures) through the school’s online platform. However, parents were not required to respond, as was used in one of the options to obtain consent described in Pokorny et al. (2001), which resulted in a response rate of 85%. Yet, digital methods also tend to elicit higher response rates from parents compared to traditional paper-based approaches, likely due to their greater convenience and accessibility (Gideon, 2012). In addition, the fact that the current study was part of an ongoing, community-based project, may also have contributed to the relatively higher consent rates. Community-members, including parents, were informed about the project and its aims (Koning et al., 2021). All in all, in line with previous studies (Anderman et al., 1995; Dent et al., 1993; Liu et al., 2017; Villarreal et al., 2023), the shift from passive to active parental consent has a major impact on the participation rate of youth in research on, in this case, alcohol use.

Considering the composition of the group adolescents that did receive parental consent to participate in this alcohol prevention study, mixed results were found. Overall, the hypothesis that at-risk adolescents were less likely to participate in the study (i.e., obtaining active consent) was rejected. Though most studies demonstrated that at-risk groups were less likely to participate in research where active consent was required (e.g., Anderman et al., 1995; Esbensen et al., 1999; Kearney et al., 1983; Pokorny et al., 2001; Tigges, 2003), significantly more adolescents in the non-consent group were older, female and somewhat higher educated. This is not a typically higher risk group as boys and lower educated are more likely to use substances compared to girls and higher educated, respectively (Inchley et al., 2020). Older adolescents were more likely to not participate because they were allowed to decide this themselves as youth older than 16 do not require parental consent.

At first sight, factors predicting non-consent may seem not in agreement in terms of level of at-risk youth. That is, adolescent girls and adolescents who perceive to have more peers that drink alcohol on a weekly basis, who perceive less strict rules about alcohol and who report higher relatedness with parents are more likely to have no active parental consent. Yet, two different mechanisms may play a role here; protective mechanism versus autonomy mechanisms. First, females and adolescents with more relatedness with their parents were less likely to participate. Overall, for general parenting, previous research has shown that parents do not parent differently across gender (Endendijk et al., 2016). Yet, when looking at behavior in relation to risk behaviors such as alcohol use, research does show that girls perceive their parents to be more strict about alcohol use than boys (Koning et al., 2022; Mehanović et al., 2022; Trager et al., 2023). In addition, the family social capital theory (Coleman, 1988) suggests that strong family bonds and relationships (i.e., relatedness) can contribute to various forms of support and resources, including protection and guidance. Parents in the current study were specifically asked to provide consent for an alcohol-prevention study, thus measuring alcohol use and related factors. Parents of girls and those who have a better relationship with their child may have wanted to “protect” the child from answering these questions. This assumption is supported by the findings that the level of autonomy was not predictive of non-consent among adolescents and that stricter alcohol-specific rules were actually the strongest predictor of non-consent.

This brings us to the second mechanism that may have taken place, that is, the autonomy mechanism. Adolescents who were older and those with more tolerant parents towards alcohol use were more likely to not participate at follow-up. It is plausible that (1) these adolescents mostly have reached the age of 16 and were therefore allowed to not participate in class if they didn’t want to. Also, the level of strictness about alcohol is declining by age of the adolescent (Koning et al., 2013). Related to the decline in strictness, we discuss the second (2) plausible mechanism (i.e., autonomy), which refers to the finding that older adolescents and those with more tolerant parents were more likely to not participate at follow-up. In line with the self-determination theory (Joussemet et al., 2008; Ryan & Deci, 2000), it is well established that an increasing level of autonomy granting/supportive parenting towards older kids is beneficial for youth development and substance use in specific (Koning et al., 2013, 2014; Mallett et al., 2011). Moreover, parents who are less strict about drinking have more tolerant attitudes about drinking (Koning et al., 2011), and are less controlling overall (Mallett et al., 2011; Moore et al., 2010; Van Zundert et al., 2006). Subsequently, these parents may be more likely to provide consent as they may not consider this topic of research as an issue, that is, may be uncertain or have a lack of interest to their child’s participation (cf. Jungmann et al., 2023; Mallett et al., 2011) or encourage their child to take responsibility for their own behavior (cf. Jungmann et al., 2023; Mynttinen et al., 2017). Though the level of autonomy perceived by adolescents was not a significant predictor of obtaining parental consent, we did observe a somewhat lower mean level of autonomy between the consent (M = 4.89, SD = 1.06) and the non-consent (M = 5.03, SD = 1.10) groups. The assumption is that adolescents in the non-consent group were provided more autonomy by their parents to make their own decision whether or not to participate (among 16+ youth) or did not feel it to be acceptable to determine this on behalf of their underage child. So far, the existence of these mechanisms are speculative and need to be further explored in future research.

This study is one of the first that examined the impact of factors on the sample composition of underaged youth due the shift from passive to active parental consent in a longitudinal and experimental design. The study involved a large sample of underaged (<18 years) adolescents and included a variety of factors across different domains relevant for adolescent development. Yet, these strengths and the findings should be considered in light of some limitations. First, this study is conducted in one community based in the Netherlands, which limits generalizability to other communities and parts in the world. Second, though the change from passive to active consent is a major strength, it may also have influenced parents’ provision of consent. That is, parents were already aware of the study as it was going on already for 2 years. On one hand, parents in support of the broader intervention (LEF) may provide active consent more easily, whereas on the other hand parents against the intervention may have had a reason to not provide this. The reasons for not providing consent do play a role and should be taken into account in future studies. Third, adolescents who did not participate at follow-up could have made this decision themselves or their parents did not provide active consent. We did not distinguish between these groups of non-consent which may be the reason why an older age was predictive of non-consent. In future studies, researchers may differentiate between these two types of non-consent (self and parent).

### Conclusion

Overall, it seems that parents do not provide active consent for different reasons. The findings suggest two distinct mechanisms that may underly non-consent: a protective mechanism, where parents—particularly of girls or those with strong parent–child bonds—may withhold consent to shield their child from sensitive topics; and an autonomy mechanism, where adolescents might independently choose not to participate. In this study, the pattern of predictors—especially the role of strict alcohol-specific parenting—points more strongly to parental protection than to adolescent autonomy.

Apart from the study specific factors investigated in the current paper, there are also practical reasons (Jungmann et al., 2023) and topic-related reasons playing that may have played a role. For example, parents may forget to sign and hand in the consent form, without any intent to resist participation of their child. This is supported by the higher rates of passive parental consent (>95%) compared to active parental consent (30–60%). This shows that about 5% of the parents really do not want their child to participate in a particular study. Also, the topic under investigation may not have directly been perceived by parents to benefit their child, which is an important factor for parents to provide consent (Van Stuijvenberg et al., 1998). As applying active parental consent is the only way to conduct research among youth, more research should be conducted on the reasons for and against providing active consent to let their child participate in research so that consent procedures can be better adapted to the values and interests based on what is important to parents (Kraft et al., 2017). Particularly, lowering the barriers for parents to provide consent seem to be a more effective way to obtain parents’ consent than increasing their benefits (Jungmann et al., 2023). In addition, alternative ways for obtaining consent should be considered, such as passive or self-consent models which may be implemented when information is age-appropriate, the process is low-stress, and the study poses minimal risk (APA, 2018). Ultimately, this is imperative for scientists to obtain representative youth samples in research. Until then, researchers should remain aware that the use of active parental consent does have an impact on the composition of the youth sample under investigation.
